# Genetically Predicted Gut Microbiota Mediate the Association Between Fatty Acids and Intrahepatic Cholestasis of Pregnancy: A Mendelian Randomization Analysis

**DOI:** 10.1002/fsn3.4683

**Published:** 2024-12-30

**Authors:** Ling Li, Zhiquan Qin, Ruirui Dong, Xiong Yuan, Gaoying Wang, Rong Wang, Shaokai Ning, Jing Wang, Jianyi Gao, Xiaoxue Tian, Ting Zhang

**Affiliations:** ^1^ Affiliated Women's Hospital of Jiangnan University, Jiangnan University Wuxi China

**Keywords:** fatty acids, gut bacteria, intrahepatic cholestasis of pregnancy, Mendelian randomization

## Abstract

Fatty acids (FAs) and gut bacteria likely play vital roles in intrahepatic cholestasis of pregnancy (ICP). However, the causal connection between FAs, gut microbiota, and ICP has not yet been confirmed. To investigate the associations of FAs, gut bacteria, and ICP, a Mendelian randomization (MR) analysis with two samples was performed to identify the potential causal relationships between FAs and ICP. The potential mediating role of gut bacteria in FAs and ICP was analyzed by a two‐step MR analysis. False discovery rate (FDR) correction was conducted to correct the bias of multiple tests. MR analysis revealed that higher omega‐6 FAs/total FAs (odds ratio [OR] = 2.563, 95% confidence interval [CI] = 1.362–4.824, FDR *p* = 0.016), linoleic acids/total FAs (OR = 3.812, 95%CI = 1.966–7.388, FDR *p* = 0.001), and average number of methylene groups (OR = 1.968, 95%CI = 1.390–2.785, FDR *p* = 0.001) are potential risk factors for ICP. However, omega‐3 FAs (OR = 0.587, 95%CI = 0.394–0.874, FDR *p* = 0.031) and the average number of double bonds in an FA chain (OR = 0.575, 95%CI = 0.435–0.759, FDR *p* = 0.001) could reduce the risk of ICP. The abundance of 25 gut bacteria showed significant causal effects on ICP, among which *Dokdonella* may play a crucial role in modulating the effects of FAs on ICP. Our research results suggest that the effects of FA on ICP likely vary according to their different types. *Dokdonella* abundance plays a significant role in mediating the causal interactions between FAs and ICP.

## Graphical abstract

The effects of fatty acids (FAs) on intrahepatic cholestasis of pregnancy (ICP) likely vary according to their different types. Dokdonella abundance plays a significant role in mediating the causal interactions between FAs and ICP.

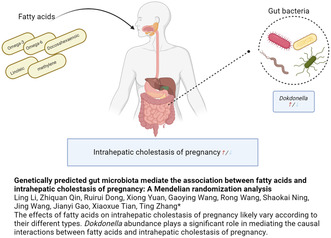

**Abbreviations**
ALA: alpha linolenic acid
DHA: docosahexaenoic acid
EPA: eicosapentaenoic acid
FAs: fatty acids
GWAS: genome‐wide association studies
ICP: intrahepatic cholestasis of pregnancy
IVs: instrumental variables
IVW: inverse variance weighting
MR: Mendelian randomization
SFAs: saturated fatty acids
SNP: single nucleotide polymorphism
USFAs: unsaturated fatty acids

## 1 Introduction

Pregnancy‐related liver illness is known as intrahepatic cholestasis of pregnancy (ICP), which is characterized by high levels of bile acids (Ibrahim et al. 2020). ICP is typically highly correlated with a variety of unfavorable consequences for both mothers and fetuses, including premature birth, stillbirth, meconium contamination, and intrauterine distress (Ibrahim et al. 2020; Monrose et al. 2021). Therefore, early detection and prevention of ICP are essential for pregnant women (Manzotti 2021). Abnormalities in lipid metabolism are important for understanding the pathogenesis and consequences of ICP during pregnancy (Chappell et al. 2012). Nevertheless, the fundamental workings of ICP remain poorly understood.

It has been demonstrated that fatty acids (FAs), an essential dietary component, are crucial in controlling a variety of physiological processes in vivo, including lipid metabolism (Chen et al. 2022). FAs are classified as unsaturated fatty acids (USFAs) or saturated fatty acids (SFAs) based on the presence or absence of a double strand (Joshi et al. 2024). Recent studies show that patients with ICP may display a different profile of FA expression than pregnant women in good health (Chen et al. 2022; Liu et al. 2022). For example, patients with ICP exhibited reduced levels of acetic acid, propionic acid, butyric acid, isovaleric acid, valeric acid, caproic acid, and greater amounts of isobutyric acid (Chen et al. 2022). Notably, elevated levels of long‐chain SFAs are correlated with an important aspect of ICP, which is an increase in bile acids (Liu et al. 2022). These investigations shed more light on the possible roles of FAs in ICP and therefore merit further research.

Many studies have shown that patients with ICP may have a different gut microbial composition than healthy individuals (Zhan et al. 2021; Li et al. 2020). For instance, Zhan et al. used 16S ribosomal RNA gene sequencing to identify that the severe ICP group had a higher abundance of the genera *Escherichia, Shigella*, *Olsenella*, and *Turicibacter* (Zhan et al. 2021). Li et al. also found that *Blautia* and *Citrobacter* were highly abundant in ICP patients (Li et al. 2020). In addition, a recent Mendelian randomization (MR) investigation demonstrated the causal connection between ICP and gut microorganisms (Li, Li, et al. 2023). Importantly, Tang et al. also found that *Bacteroides fragilis* in individuals with ICP could elevate ICP by blocking farnesoid × receptor signaling through its bile salt hydrolase activity to regulate bile acid metabolism (Tang et al. 2023). These significant results highlight the potential involvement of gut bacteria in the onset and progression of ICP. Furthermore. The transition from long‐chain FAs to short‐chain FAs by gut bacteria is an essential component of FA metabolism (Jia et al. 2024). However, the connection between FAs and gut microbiota in ICP patients has received minimal investigation.

Mendelian randomization is a genetic epidemiology technique that evaluates the causal links between different features. This method evaluates the causal importance of features in the development of disease by using genetic variation as a substitute for traits within an instrumental variable framework (Laskar et al. 2024). Compared to traditional analyzes carried out in an observational setting, this method is less susceptible to bias and confounding, because germline genetic variation is theoretically random between generations and fixed at conception (Laskar et al. 2024; Martin et al. 2024). Mendelian randomization has long been used to investigate how exposure affects the course of diseases (Martin et al. 2024). For example, Wang et al. discovered that USFAs, particularly monounsaturated FAs, may have a protective effect against preeclampsia after using MR to examine the unsubstantiated connections between USFAs and the condition (Wang et al. 2024).

To identify whether the correlations between FAs and ICP are causal, we used a two‐sample MR analysis in this work, using inverse variance weighted (IVW), Wald ratio, MR‐Egger, and weighted median approaches. A two‐step MR analysis was also conducted to detect the potential role of gut bacteria in FAs and ICP. This MR investigation has the potential to offer proof of a hereditary causal relationship between FAs and ICP, thus providing further avenues for potential diet therapy in ICP.

## 2 Materials and Methods

### 2.1 Study Design

**FIGURE 1:**
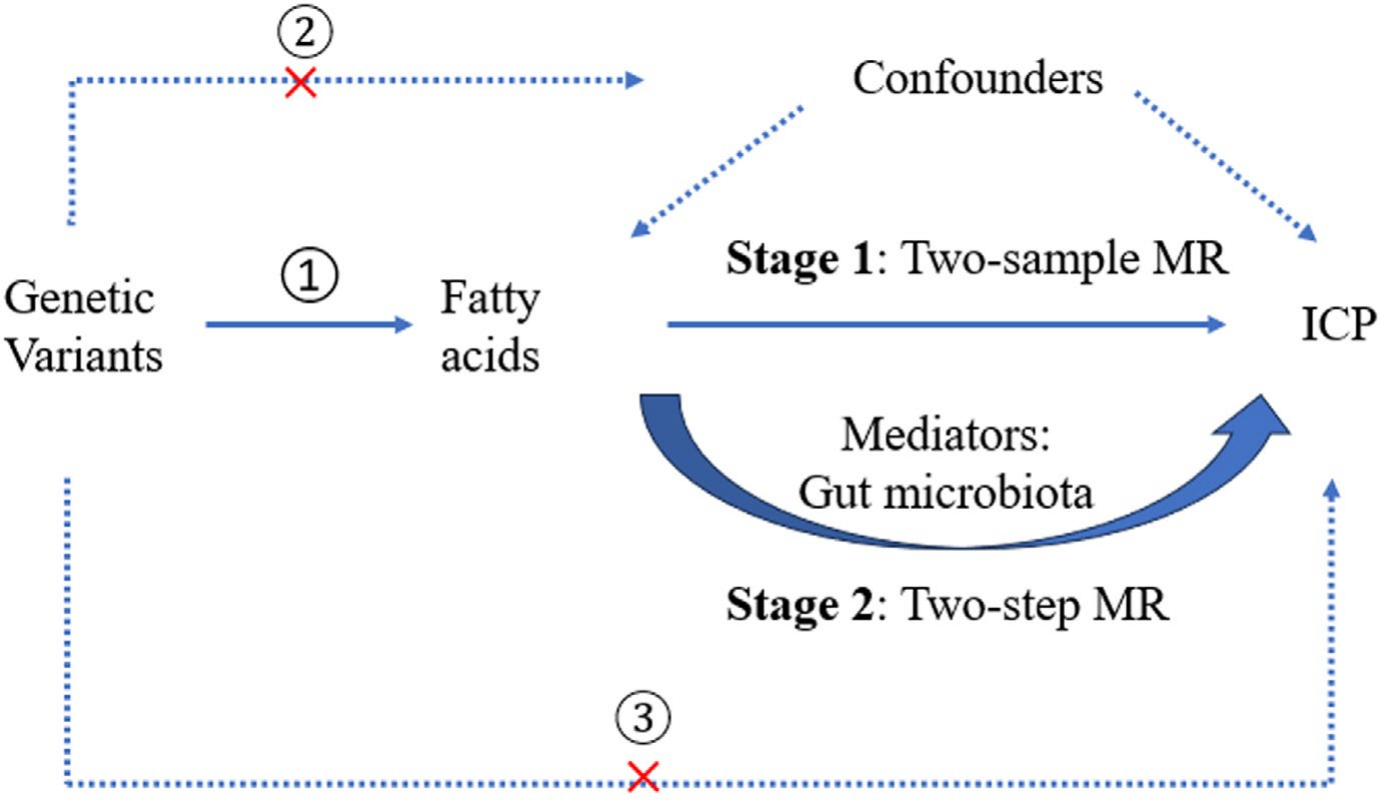
Design of this MR study.

The study design is presented in Figure 1. First, we employed two‐sample MR to identify the causal relationships between FAs (totals FA, FA characteristics, and individual FAs) and ICP. Next, we detected the correlation between gut bacteria and ICP. Last, we determined if the abundance of meaningful gut bacteria was influenced by FAs associated with ICP.

### 2.2 Data Sources

The exposure data including gut bacteria; total FAs; traits of FAs, such as degree of unsaturation measurement and average number of double bonds in an FA chain; and polyunsaturated FAs including omega‐3, omega‐3/total FAs, omega‐6, omega‐6/total FAs, omega‐6/omega‐3, docosahexaenoic acid (DHA), DHA/total FAs, linoleic acid, and linoleic acid/total FAs were obtained from genome‐wide association studies (GWAS). The outcome data was acquired from the largest GWAS meta‐analysis of ICP conducted by Dixon et al. The data of gut bacteria was acquired from GWAS analysis of 5959 individuals enrolled in the FR02 cohort, including a total of 473 distinct Genome Taxonomy Database (GTDB) taxa, which represented 17% of all tested taxa and included 11 phyla, 19 classes, 24 orders, 62 families, 146 genera, and 209 species. All the populations of the GWAS data were European. The details of GWAS data used in this study are presented in Table S1.

### 2.3 Single Nucleotide Polymorphism (SNP) Selection

Three fundamental presumptions were maintained to guarantee the validity of our MR analysis: (i) the genetic variants selected should exhibit strong correlations with the exposure of interest; (ii) the genetic variants utilized should not have any effect on the outcome other than that which results from the selected exposure; and (iii) the genetic variants should not have any correlations with any confounding factors that could alter the relationship between the two variables.

First, SNPs that were significantly related to FAs (*p* < 5 × 10^−8^) were chosen as instrumental variables (IVs). For the data of gut bacteria, SNPs (*p* < 1 × 10^−5^) were selected to ensure sufficient IVs. After that, SNP trimming was done within a 10,000 kb window size to make sure that the *r* ^2^ < 0.001 threshold did not exceed the linkage disequilibrium between SNPs. Subsequently, SNPs with F‐statistic > 10 were selected for further investigation to guard against weak instrument bias. Additionally, palindromic SNPs were eliminated. Tables [Link], [Link], [Link] present a list of every SNP used in this investigation.

### 2.4 MR Analysis

Following recognized approaches, the R package TwoSample MR (version 0.5.8) was used to comprehensively assess the causative effects. The evaluation of causal effects was the primary use of the IVW approach. When all of the SNPs in the MR analysis are legitimate IVs, the IVW approach can be used. We employed the Wald ratio as the primary technique to evaluate causal effects for exposures to a single relevant SNP as the IV. Additionally, weighted median and MR‐Egger methods were applied in addition to IVW. Three fundamental assumptions are met by the weighted median approach, which is based on the idea that at least 50% of the genetic diversity is legitimate. The MR‐Egger method takes into account the existence of the intercept term that can be used as an addition to the IVW test, but may be biased and inflate the type I error, and assumes that > 50% of genetic variation is incorrect (i.e., does not follow the three basic assumptions). The Cochran's *Q* test and MR‐PRESSO were utilized to assess heterogeneity between the two sets of data.

## 3 Statistical Analysis

### 3.1 Results

In this investigation, the two‐sample MR package (version 0.5.8) was employed. The MR‐PRESSO analysis was carried out using the MR‐PRESSO package. A *p* value < 0.05 was regarded as possibly causative evidence. When the FDR *p* value was < 0.05, a significant difference was considered.

### 3.2 Study Design

Figure 1 shows an overview of our study design. First, we used two‐sample MR to identify the causal relationships between FAs (totals FA, FA characteristics, and individual FAs) and ICP. Next, we detected the correlation between the abundance of gut bacteria and ICP. Last, we determined if the abundance of meaningful gut microbiota mediated the links between FAs and ICP. Methods of IVW, Wald ratio, weighted median, and MR‐Egger were employed in the MR analysis. Sensitivity analysis was also conducted, including MR‐PRESSO and Cochran's *Q* test. Tables [Link], [Link], [Link] present the SNPs used for MR analysis in the causal effects of FAs on ICP, FAs on gut bacteria, and gut bacteria on ICP.

The flowchart of the study's three basic hypotheses is as follows: (1) There is a strong correlation between instrumental factors and exposure; (2) there is no correlation between instrumental variables and confounders that affect the relationship between exposure and outcome; and (3) there is a correlation only between instrumental variables and outcome through exposure.

### 3.3 Causal Effects of FAs and Traits of FAs on ICP

**FIGURE 2:**
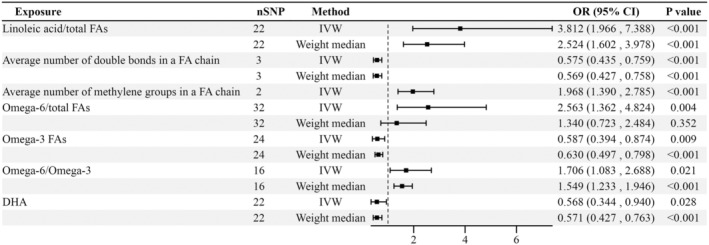
Forest plot to visualize the causal effects of FAs and traits of FAs on ICP.

Figure 2 depicts the MR methods to ascertain the causal relationships between FAs and their traits on ICP. According to the IVW and Wald ratio results, higher omega‐6/omega‐3 (odds ratio [OR] = 1.706, 95% confidence interval [CI] = 1.083–2.688, *p* = 0.021) is a potential risk for ICP, whereas DHA (OR = 0.568, 95%CI = 0.344–0.940, *p* = 0.028) may exert a protective effect against ICP. After FDR correction, the average number of double bonds in a fatty acid chain (OR = 0.575, 95%CI = 0.435–0.759, FDR *p* = 0.001) and omega‐3 FAs (OR = 0.587, 95%CI = 0.394–0.874, FDR *p* = 0.031) could lower the risk of ICP. However, the risk of ICP may increase if the average number of methylene groups (OR = 1.968, 95%CI = 1.390–2.785, FDR *p* = 0.001), linoleic acids/total FAs (OR = 3.812, 95%CI = 1.966–7.388, FDR *p* = 0.001), and omega‐6 FAs/total FAs (OR = 2.563, 95%CI = 1.362–4.824, FDR *p* = 0.016) increases.

Pleiotropy was not found in our study. For positive results and existing heterogeneity, we also used the method of weighted median to evaluate the causal links. The results were consistent with the previous results of IVW. It is worth noting that the result of omega‐6/total FAs (OR = 1.340, 95%CI = 0.723–2.484, *p* = 0.352) on ICP was not significant according to the weighted median method. Among the IVs, no statistically significant pleiotropic effects were found, as confirmed by the MR‐Egger intercept approach.

### 3.4 Causal Effects of Gut Bacteria on ICP

**FIGURE 3:**
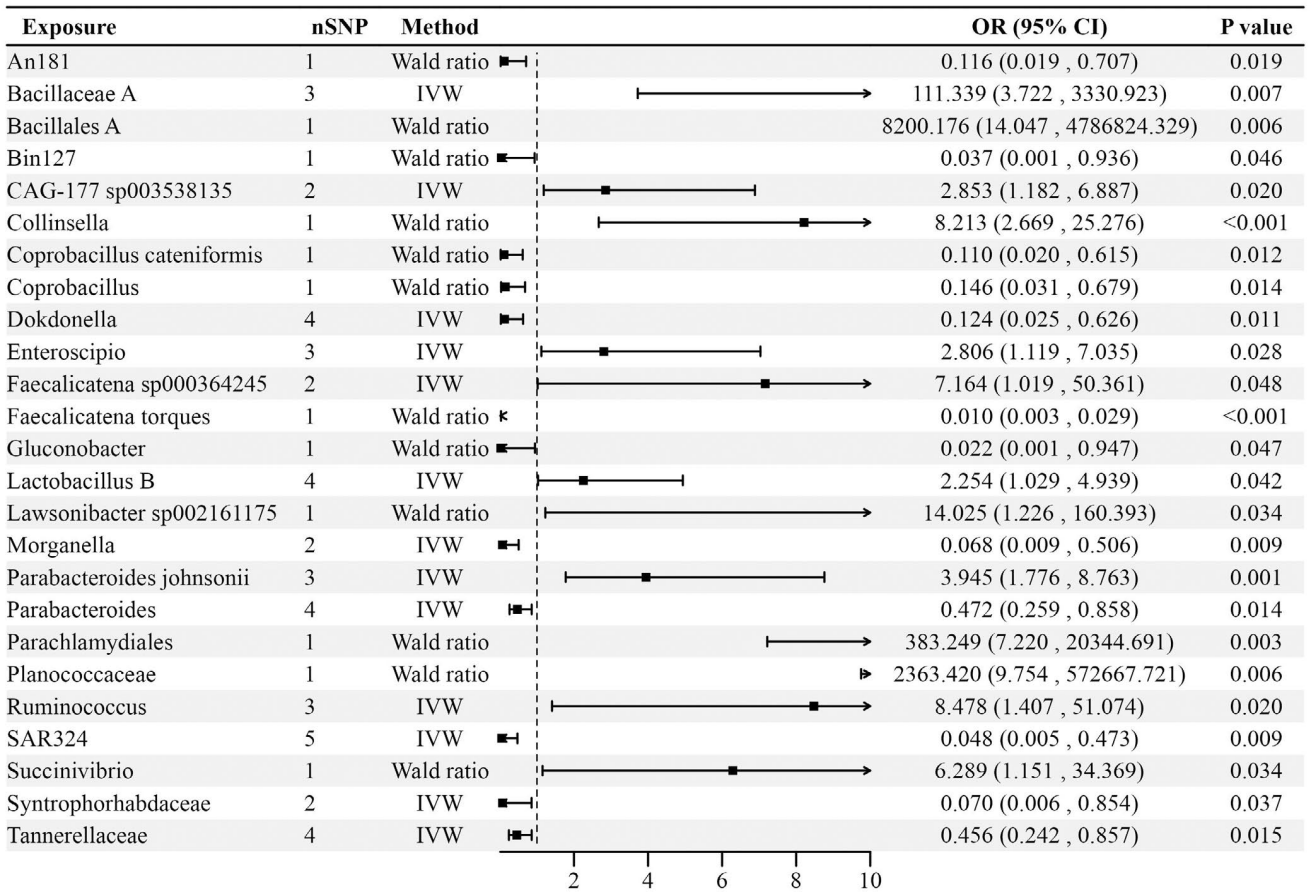
Forest plot to visualize the causal effects of gut bacteria on ICP.

Furthermore, we detected the causal effects of gut bacteria on ICP (Figure 3). MR analysis showed that the abundance of Bacillales A (OR = 8200.176, 95%CI = 14.047–4786824.33, *p* = 0.006); *Planococcaceae* (OR = 2363.42, 95%CI = 9.754–572667.721, *p* = 0.006); *Parachlamydiales* (OR = 383.249, 95%CI = 7.220–20344.691, *p* = 0.003); *Bacillaceae A* (OR = 111.339, 95%CI = 3.722–3330.923, *p* = *0,007*); *Lawsonibacter* sp002161175 (OR = 14.025, 95%CI = 1.226–160.393, *p* = 0.034); *Ruminococcus* (OR = 8.478, 95%CI = 1.407–51.074, *p* = 0.020); *Collinsella* (OR = 8.213, 95%CI = 2.669–25.276, FDR *p* = 0.041); *Faecalicatena* sp000364245 (OR = 7.164, 95%CI = 1.019–50.361, *p* = 0.048); *Succinivibrio* (OR = 6.289, 95%CI = 1.151–34.369, *p* = 0.034); *Parabacteroides johnsonii* (OR = 3.945, 95%CI = 1.776–8.763, *p* = 0.001); CAG‐177 sp003538135 (OR = 2.853, 95%CI = 1.182–6.887, *p* = 0.020); *Enteroscipio* (OR = 2.806, 95%CI = 1.119–7.035, *p* = 0.028); and *Lactobacillus B* (OR = 2.254, 95%CI = 1.029–4.939, *p* = 0.042) were positively associated with higher risk of ICP. We also found that *Parabacteroides* (OR = 0.472, 95%CI = 0.259–0.858, *p* = 0.014); *Tannerellaceae* (OR = 0.456, 95%CI = 0.242–0.857, *p* = 0.015); *Coprobacillus* (OR = 0.146, 95%CI = 0.031–0.679, *p* = 0.014); *Dokdonella* (OR = 0.124, 95%CI = 0.025–0.626, *p* = 0.011); An181 (OR = 0.116, 95%CI = 0.019–0.707, *p* = 0.019), *Coprobacillus cateniformis* (OR = 0.110, 95%CI = 0.020–0.615, *p* = 0.012); *Syntrophorhabdaceae* (OR = 0.070, 95%CI = 0.006–0.854, *p* = 0.037); *Morganella* (OR = 0.068, 95%CI = 0.009–0.506, *p* = 0.009); SAR324 (OR = 0.048, 95%CI = 0.005–0.473, *p* = 0.009); Bin127 (OR = 0.037, 95%CI = 0.001–0.936, *p* = 0.046); *Gluconobacter* (OR = 0.022, 95%CI = 0.001–0.947, *p* = 0.047); and *Faecalicatena torques* (OR = 0.010, 95%CI = 0.003–0.029, FDR *p* = 2.204 × 10^−13^) could decrease the occurrence of ICP. We did not find any heterogeneity or pleiotropy in these results.

### 3.5 The Mediating Role of Gut Bacteria in the Causal Link Between FAs and ICP

**FIGURE 4:**
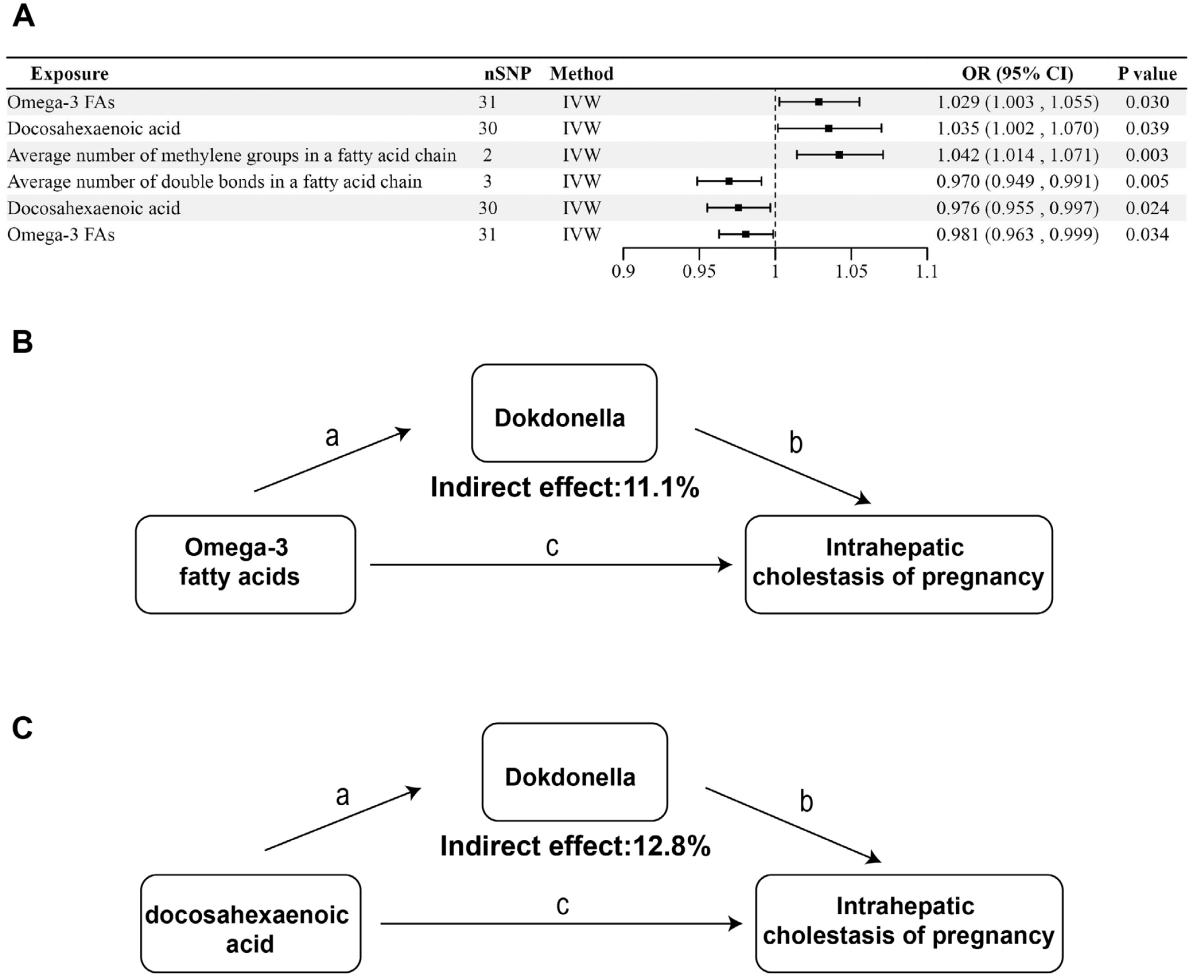
Forest plot and schematic diagrams to visualize the mediating role of gut bacteria in the causal link between FAs and ICP.

We then detected the mediating role of beneficial gut bacteria in causal effects between FAs (Figure 4). The abundance of *Dokdonella* was upregulated by omega‐3 FAs (OR = 1.029, 95%CI = 1.003–1.055, *p* = 0.030) and downregulated by DHA (OR = 1.035, 95%CI = 1.002–1.070, *p* = 0.039) (Figure 4A). *Dokdonella* showed mediating roles in FAs on ICP, including omega‐3 FAs (effect proportion = 11.1%) (Figure 4B) and DHA (effect proportion = 12.8%) (Figure 4C). Besides, the abundance of SAR324 was upregulated by the average number of methylene groups in an FA chain (OR = 1.042, 95%CI = 1.014–1.071, FDR *p* = 0.003) and downregulated by the average number of double bonds in an FA chain (OR = 0.970, 95%CI = 0.949–0.991, FDR *p* = 0.019); DHA (OR = 0.976, 95%CI = 0.955–0.997, *p* = 0.024); and omega‐3 FAs (OR = 0.981, 95%CI = 0.963–0.999, *p* = 0.034) (Figure 4A). However, SAR324 did not directly mediate the causal effect of FAs on ICP.

## 4 Discussion

In this study, we used MR to detect the mediating effects of gut microbiota in FAs on ICP. These results revealed the following: (1) Higher omega‐6 FAs/total FAs, omega‐6/omega‐3, linoleic acids/total FAs, and an average number of methylene groups are potential risk factors for ICP. However, omega‐3 FAs, DHA, and the average number of double bonds in an FA chain could reduce the risk of ICP; (2) The abundance of 25 gut bacteria showed significant causal effects on ICP, including 13 pathogenic and 12 protective microorganisms; and (3) *Dokdonella* may play a significant role in modulating the effects of FAs on ICP.

Omega‐3 FAs consist of DHA, alpha‐linolenic acid (ALA), and eicosapentaenoic acid (EPA). Common foods that contain high concentrations of omega‐3 FAs include fatty fish, shellfish, cereals, seeds, nuts, and vegetables (Liu, Shan, and Rehman 2024; Luo et al. 2024). Pregnant women with low total omega‐3 FAs may have an increased risk of early preterm birth (Simmonds et al. 2020). Besides, supplements of omega‐3 FAs during pregnancy could considerably benefit both the mother and fetus. For example, Cetin et al. recommended a supply of at least 250 mg/day DHA + EPA for women of gestational age, as well as an additional intake of 100–200 mg/day for the mother, which could lower the risk of preterm birth (Cetin et al. 2024). Omega‐3 FAs are usually considered to exert anti‐inflammatory effects (Chávez‐Ortega et al. 2024; Xie et al. 2024). In a meta‐analysis of randomized controlled trials, Xie et al. found that omega‐3 could greatly reduce the level of serum C‐reactive protein, a biomarker of inflammation (Xie et al. 2024). Another study demonstrated that ample supplements of EPA + DHA could promote the synthesis of muscle protein by decreasing inflammation (Blaauw et al. 2024). As a significant component of omega‐3 FAs, DHA is frequently prescribed to ensure healthy fetal development during gestation, because it plays a significant function in fetal neurodevelopment (Moltu et al. 2024). Besides, DHA supplementation increases birth weight and decreases the risk of preterm birth and neonatal intensive care unit (NICU) hospitalization (Wang et al. 2023). Further, previous MR analyzes have found that DHA may reduce the risks of many diseases such as idiopathic normal pressure hydrocephalus (Li, Huang, et al. 2023) and pregnancy‐induced hypertension (Ma and He 2023). However, few studies have investigated the link between DHA and ICP. Our research showed that high DHA levels may protect against ICP, suggesting that DHA‐centered diet therapy is a promising treatment option.

Omega‐6 FAs, unlike omega‐3 FAs, are typically considered pro‐inflammatory FAs (Sanchez et al. 2024; Broos et al. 2024). Through MR analysis, omega‐6 FAs have been identified as risk factors for many diseases such as psoriasis (Li et al. 2024), bipolar disorder (Stacey et al. 2024), and chronic kidney disease (Huang et al. 2023). Interestingly, omega‐6 FAs also reduce the incidence of many diseases like knee and hip osteoarthritis (Li et al. 2023) and, background and proliferative diabetic retinopathy (Ren et al. 2023). Nevertheless, little study has been carried out on the connections between ICP and omega‐6. In our study, higher omega‐6/total FAs and omega‐6/omega‐3, instead of total omega‐6 FAs levels, could play a role in promoting the occurrence of ICP, but this needs to be verified in a larger cohort.

Early research revealed that gut microbiota plays a crucial role in the development of ICP (Zhan et al. 2021). Li et al. found a decreased *Ruminococcaceae* abundance in ICP patients, which contributed significantly to the metabolism of hypoxanthine in ICP (Li, Xie et al. 2023). Another research also found a dysbiosis of *Ruminococcus* in ICP rats (Li et al. 2022). The torques group of the genus *Ruminococcus* was also found to decrease the risk of ICP in one MR analysis (Li, Li et al. 2023). However, relevant studies relating to our positive MR results are still lacking.

Our research has the following strengths. To our knowledge, this is the first study to systematically investigate the causal effects of FAs and ICP. Second, we found that various FAs may control the number of *Dokdonella* and SAR324, and *Dokdonella* may affect the prevalence of ICP.

Our study also has some limitations. First, the results of our study only apply to the European population owing to the limited availability of data. Furthermore, given that both the exposure and result datasets come from European populations, there may be some sample overlap. To establish the presence of a cause‐and‐effect link, it is critical to investigate and validate this causal inference utilizing RCTs with high certainty and more stringent control.

## 5 Conclusions

This MR study discovered that DHA, omega‐3, and FA chains with a higher average number of double bonds prevent ICP. However, linoleic acid/total FAs, omega‐6/omega‐3, omega‐6/total FAs, and the average amount of methylene groups may all increase the risk of ICP. Furthermore, numerous FAs may govern the quantity of *Dokdonella*, thereby influencing the risk of ICP. Significantly, these findings suggest that pregnant women's FA intake should be monitored to develop treatment strategies for ICP avoidance.

## Author Contributions

**Ling Li:** conceptualization (equal), data curation (equal), formal analysis (equal), funding acquisition (equal), investigation (equal), methodology (equal), resources (equal), software (equal), validation (equal), visualization (equal), writing – original draft (equal), writing – review and editing (equal). **Ting Zhang:** conceptualization (lead), funding acquisition (lead), project administration (lead), resources (lead), supervision (lead), writing – review and editing (equal). **Zhiquan Qin:** data curation (equal), formal analysis (equal), investigation (equal), methodology (equal), resources (equal), software (equal), validation (equal), visualization (equal), writing – review and editing (equal). **Ruirui Dong:** formal analysis (equal), investigation (equal), methodology (equal), validation (equal), writing – review and editing (equal). **Xiong Yuan:** formal analysis (equal), investigation (equal), methodology (equal), validation (equal), writing – review and editing (equal). **Gaoying Wang:** writing – review and editing (supporting). **Rong Wang:** writing – review and editing (supporting). **Shaokai Ning:** writing – review and editing (equal). **Jing Wang:** writing – review and editing (supporting). **Jianyi Gao:** writing – review and editing (supporting). **Xiaoxue Tian:** writing – review and editing (supporting).

## Ethics Statement

Here, our study is based on the large‐scale GWAS datasets, and not the individual‐level data. The studies included in these consortia obtained approval from local research ethics committees and institutional review boards, and all participants provided written informed consent.

## Consent

The authors have nothing to report.

## Conflicts of Interest

The authors declare no conflicts of interest.

## Supporting information


**Table S1.** Data included in this study.

**Table S2.** SNPs used for MR analysis in FAs and ICP.

**Table S3.** SNPs used for MR analysis in gut bacteria and ICP.

**Table S4.** SNPs used for MR analysis FAs and gut bacteria.

## Data Availability

All data is obtained from OpenGWAS (https://gwas.mrcieu.ac.uk/) and GWAS (https://www.ebi.ac.uk/gwas/).
