# Supplementary material for: Genetically Predicted Gut Microbiota Mediate the Association Between Fatty Acids and Intrahepatic Cholestasis of Pregnancy: A Mendelian Randomization Analysis
Source: Food Sci Nutr. 2024 Dec 30;13(1):e4683. doi: 10.1002/fsn3.4683 (PMC11717022; doi:10.1002/fsn3.4683)
Supplement: Supplementary file 1 — Table S1. Data included in this study. [file FSN3-13-e4683-s002.pdf]

Supplemental Table 1 The data information in this study

**Exposure**	**GWAS id**	**Sample size**	**Population**
Total FAs	ebi-a-GCST90092987	115,006	European
Omega-3 FAs	ebi-a-GCST90092931	115,006	European
Omega-3/Total FAs	ebi-a-GCST90092932	115,006	European
Omega-6 FAs	ebi-a-GCST90092933	115,006	European
Omega-6/Total FAs	ebi-a-GCST90092935	115,006	European
Omega-6/Omega-3	ebi-a-GCST90092934	115,006	European
DHA	ebi-a-GCST90092816	115,006	European
DHA/total FAs	ebi-a-GCST90092817	115,006	European
Linoleic acid	ebi-a-GCST90092880	115,006	European
Linoleic acid/total FAs	ebi-a-GCST90092881	115,006	European
Other polyunsaturated fatty acids than 18:2	met-c-917	13.549	European
Saturated FAs	ebi-a-GCST90092980	115,006	European
Monounsaturated FAs	ebi-a-GCST90092928	115,006	European
Polyunsaturated FAs	ebi-a-GCST90092939	115,006	European
Polyunsaturated FAs/total FAs	ebi-a-GCST90092941	115,006	European
Degree of unsaturation	ebi-a-GCST90092994	115,006	European
Average number of double bonds in a fatty acid chain	met-c-851	15,728	European
Average number of methylene groups in a fatty acid chain	met-c-848	19,021	European
Gut bacteria	GCST90032172 - GCST90032644	5959	European

**Outcome**	**GWAS id**	**No. of cases**	**No. of controls**	**Population**
Intrahepatic cholestasis of pregnancy (ICP)	GCST90095084	1138	153642	European
