# Supplementary material for: Genetically Predicted Gut Microbiota Mediate the Association Between Fatty Acids and Intrahepatic Cholestasis of Pregnancy: A Mendelian Randomization Analysis
Source: Food Sci Nutr. 2024 Dec 30;13(1):e4683. doi: 10.1002/fsn3.4683 (PMC11717022; doi:10.1002/fsn3.4683)
Supplement: Supplementary file 2 — Table S2. SNPs used for MR analysis in FAs and ICP. [file FSN3-13-e4683-s001.pdf]

Supplementary Table 2: SNPs used for MR analysis in fatty acids and intrahepatic cholestasis of pregnancy

Exposure	SNP	effect allele	other allele	beta	eaf	F
	rs11122450	G	T	0.022	0.611	14.390
	rs12226389	C	T	-0.047	0.186	25.495
	rs1260326	C	T	-0.048	0.604	68.252
	rs12914626	T	C	-0.061	0.702	83.002
	rs1560390	C	T	-0.050	0.220	37.751
	rs174564	G	A	-0.290	0.347	2309.862
	rs261291	C	T	0.114	0.356	354.631
	rs273912	T	G	0.026	0.707	15.165
	rs2853968	G	A	0.025	0.403	18.107
**DHA**	rs312939	A	G	0.039	0.752	27.128
	rs3764261	A	C	0.039	0.324	37.635
	rs4860987	T	A	0.038	0.259	25.066
	rs525028	A	G	-0.030	0.709	20.038
	rs58542926	T	C	-0.120	0.074	35.310
	rs638714	T	G	-0.038	0.346	37.450
	rs660240	C	T	0.043	0.785	27.612
	rs673335	C	T	-0.057	0.160	30.450
	rs73045691	A	G	0.029	0.304	18.001
	rs7570469	T	C	-0.023	0.669	13.741
	rs7924036	T	G	0.035	0.504	39.059
	rs9304381	T	C	0.050	0.818	28.255
	rs9987289	G	A	0.056	0.909	11.317
**Exposure**	**SNP**	**effect allele**	**other allele**	**beta**	**eaf**	**F**
	rs10455872	G	A	0.082	0.079	17.997
	rs12226389	C	T	-0.046	0.186	23.933
	rs1532085	G	A	-0.058	0.614	95.507
	rs174564	G	A	-0.275	0.347	2004.627
	rs1800588	T	C	0.068	0.216	66.248
	rs195445	T	C	-0.030	0.688	20.624
	rs2081194	C	G	0.025	0.601	16.664
	rs2294919	T	C	-0.027	0.248	12.599
**DHA/total FAs**	rs272888	C	T	0.025	0.707	13.735
	rs328	G	C	0.093	0.100	35.222
	rs508049	T	C	-0.098	0.049	10.349
	rs6452788	A	G	0.027	0.240	11.778
	rs676210	A	G	0.036	0.206	17.765
	rs7924036	T	G	0.046	0.504	65.194
	rs964184	C	G	0.069	0.867	32.002
**Exposure**	**SNP**	**effect allele**	**other allele**	**beta**	**eaf**	**F**
	rs1002687	A	G	0.088	0.645	197.329
	rs12916	C	T	0.050	0.400	71.026
	rs13108218	G	A	-0.032	0.615	28.305
	rs174564	G	A	0.084	0.347	178.917
	rs2126259	C	T	0.070	0.899	19.681
	rs2389599	C	G	0.023	0.525	15.958
	rs247617	A	C	0.051	0.324	60.040
	rs261290	C	T	-0.090	0.655	198.929
**Linoleic acid**	rs2740488	C	A	-0.048	0.265	42.292
	rs3011437	G	T	0.036	0.294	26.437
	rs35350651	AC	A	0.025	0.503	19.540
	rs3817335	A	T	-0.026	0.351	17.480
	rs4299376	T	G	-0.039	0.676	35.498
	rs4665972	C	T	-0.051	0.605	72.508
	rs4939883	C	T	0.063	0.819	42.026
	rs514230	T	A	0.029	0.518	25.726
	rs55747707	A	G	-0.046	0.204	26.470
	rs58542926	T	C	-0.110	0.074	27.946
	rs602633	G	T	0.053	0.783	40.102
	rs633695	G	A	0.070	0.292	101.946
	rs6471717	A	G	-0.031	0.663	23.868
	rs6602911	T	C	0.024	0.360	15.279
	rs6882345	A	G	0.044	0.633	50.001
	rs693	A	G	0.061	0.521	111.985
	rs7707394	A	G	0.032	0.357	25.647
	rs7750288	G	A	0.027	0.285	14.544
	rs865716	T	A	-0.024	0.499	17.129
	rs964184	C	G	-0.145	0.867	136.332
**Exposure**	**SNP**	**effect allele**	**other allele**	**beta**	**eaf**	**F**
	rs10733306	T	C	0.025	0.460	19.490
	rs11030088	A	G	-0.031	0.251	17.130
	rs1260326	C	T	0.082	0.604	192.571
	rs13179413	T	C	-0.029	0.285	17.469
	rs13389219	T	C	0.028	0.393	22.955
	rs17244834	T	A	0.033	0.374	29.989
	rs174567	G	A	0.186	0.348	917.270
	rs2180725	C	T	-0.026	0.232	11.140
	rs2576362	T	G	0.027	0.255	13.288
**Linoleic acid/total FAs**	rs2835028	C	A	0.024	0.412	16.615
	rs2843126	G	A	0.024	0.518	17.450
	rs28601761	G	C	0.066	0.420	128.829
	rs2927468	G	A	0.024	0.523	17.011
	rs2972143	G	A	-0.028	0.646	20.659
	rs34062580	A	G	0.066	0.130	28.280
	rs34620476	A	C	0.031	0.432	28.623
	rs4299376	T	G	-0.028	0.676	18.434
	rs4810479	T	C	0.039	0.750	26.560
	rs58365744	C	A	-0.027	0.219	10.456
	rs6683445	A	C	-0.024	0.532	18.123
	rs7819706	G	A	0.068	0.118	25.118
	rs964184	C	G	0.044	0.867	13.071
	rs9908820	G	A	-0.025	0.729	12.674
**Exposure**	**SNP**	**effect allele**	**other allele**	**beta**	**eaf**	**F**
	rs1002687	A	G	0.091	0.645	212.684
	rs10455872	G	A	-0.148	0.079	56.454
	rs1052248	A	T	0.031	0.258	17.285
	rs10761716	G	C	-0.031	0.441	27.942
	rs1128249	T	G	-0.036	0.392	34.821
	rs11429307	GT	G	0.043	0.191	21.418
	rs117733303	G	A	-0.258	0.019	10.625
	rs11940694	G	A	0.025	0.605	17.392
	rs1260326	C	T	-0.111	0.604	342.545
	rs12916	C	T	0.023	0.400	14.589
	rs13108218	G	A	-0.038	0.615	38.381
	rs1394092	T	C	0.031	0.727	18.101
	rs1471251	T	A	0.030	0.397	24.891
	rs1540037	G	A	0.033	0.778	15.369
	rs174564	G	A	0.064	0.347	102.463
	rs1993453	G	A	-0.026	0.663	16.197
	rs2000999	A	G	0.030	0.189	10.103
	rs261290	C	T	-0.073	0.655	132.142
	rs2721961	G	T	-0.026	0.281	13.192
	rs2740488	C	A	-0.028	0.265	14.283
	rs28601761	G	C	-0.089	0.420	224.359
	rs2943635	T	C	0.026	0.680	15.888
**Monounsaturated FAs**	rs3198697	T	C	-0.027	0.407	19.939
	rs328	G	C	-0.145	0.100	82.835
	rs3812316	G	C	-0.113	0.129	78.955
	rs4564803	T	G	-0.072	0.228	77.436
	rs4704834	G	A	0.044	0.644	48.219
	rs4846914	A	G	-0.031	0.605	25.704
	rs526748	T	G	0.025	0.554	18.834
	rs602633	G	T	0.027	0.783	10.167
	rs60960031	A	G	-0.026	0.403	19.121
	rs615632	T	C	0.033	0.532	31.934

	rs632057	G	T	-0.031	0.628	26.054
	rs633695	G	A	0.056	0.292	64.718
	rs6905288	A	G	0.023	0.569	14.903
	rs7140110	C	T	0.031	0.299	20.218
	rs7679	C	T	0.037	0.186	14.924
	rs7973253	G	A	0.026	0.367	17.848
	rs8107974	T	A	-0.118	0.076	32.707
	rs964184	C	G	-0.205	0.867	270.577
**Exposure**	**SNP**	**effect allele**	**other allele**	**beta**	**eaf**	**F**
	rs10096633	T	C	-0.042	0.124	10.573
	rs10455872	G	A	-0.062	0.079	10.312
	rs12037485	T	C	0.022	0.460	15.666
	rs12226389	C	T	-0.054	0.186	33.300
	rs1260326	C	T	-0.083	0.604	202.365
	rs174564	G	A	-0.337	0.347	3100.766
	rs2187375	G	A	0.052	0.823	29.879
	rs2247056	C	T	0.036	0.703	28.301
	rs2288912	G	C	0.027	0.496	22.357
	rs261290	C	T	-0.113	0.655	332.724
**Omega-3 FAs**	rs312939	A	G	0.034	0.752	20.812
	rs4860987	T	A	0.045	0.259	34.034
	rs583609	C	T	-0.071	0.353	135.118
	rs58542926	T	C	-0.173	0.074	72.347
	rs6129624	A	G	-0.024	0.335	14.469
	rs62466318	T	C	-0.072	0.204	68.811
	rs633695	G	A	0.085	0.292	156.511
	rs660240	C	T	0.037	0.785	19.985
	rs6602911	T	C	0.023	0.360	14.587
	rs673335	C	T	-0.068	0.160	43.212
	rs6882345	A	G	0.027	0.633	20.800
	rs7924036	T	G	0.022	0.504	15.779
	rs7970695	A	G	-0.024	0.621	15.608
	rs964184	C	G	-0.118	0.867	94.528
	rs9987289	G	A	0.054	0.909	10.314
**Exposure**	**SNP**	**effect allele**	**other allele**	**beta**	**eaf**	**F**
	rs1077835	G	A	0.091	0.220	123.932
	rs12226389	C	T	-0.064	0.186	47.839
	rs1260326	C	T	-0.039	0.604	43.470
	rs1532085	G	A	-0.078	0.614	175.173
	rs174564	G	A	-0.391	0.347	4217.473
	rs195445	T	C	-0.038	0.688	34.313
	rs272888	C	T	0.028	0.707	17.539
**Omega-3/Total FAs**	rs4860987	T	A	0.039	0.259	26.078
	rs508049	T	C	-0.106	0.049	12.394
	rs58542926	T	C	-0.132	0.074	42.061
	rs61886804	G	T	0.035	0.200	16.093
	rs62466318	T	C	-0.040	0.204	21.790
	rs638714	T	G	-0.032	0.346	26.834
	rs662138	G	C	-0.031	0.186	11.386
	rs6693447	G	T	0.026	0.462	20.499
	rs6717316	A	G	-0.024	0.670	14.442
	rs7924036	T	G	0.037	0.504	44.554
	rs7944950	C	T	-0.074	0.086	17.260
	rs8074191	C	T	-0.027	0.756	12.478
	rs964184	C	G	-0.039	0.867	10.315
	rs9947684	G	A	0.028	0.654	20.828
**Exposure**	**SNP**	**effect allele**	**other allele**	**beta**	**eaf**	**F**
	rs1002687	A	G	0.092	0.645	218.036
	rs11789603	T	C	0.048	0.109	10.793
	rs1260326	C	T	-0.065	0.604	118.232
	rs13108218	G	A	-0.035	0.615	33.555
	rs2126259	C	T	0.083	0.899	27.846
	rs261290	C	T	-0.097	0.655	233.944
	rs2721961	G	T	-0.027	0.281	15.107
	rs2740488	C	A	-0.050	0.265	45.886
	rs35350651	AC	A	0.028	0.503	23.694
	rs3756772	T	C	0.023	0.401	15.423
	rs3764261	A	C	0.062	0.324	89.844
	rs3770586	T	C	-0.023	0.484	16.090
	rs3817335	A	T	-0.028	0.351	20.078
	rs4299376	T	G	-0.036	0.676	29.948
	rs4704210	C	G	0.046	0.374	57.976
**Omega-6 FAs**	rs4860948	A	T	0.029	0.244	13.647
	rs496654	C	A	0.030	0.517	28.191
	rs55747707	A	G	-0.050	0.204	31.934
	rs58542926	T	C	-0.128	0.074	38.473
	rs602633	G	T	0.057	0.783	47.083
	rs633695	G	A	0.073	0.292	111.797
	rs6471717	A	G	-0.030	0.663	21.423
	rs6602911	T	C	0.026	0.360	18.050
	rs672889	G	T	0.076	0.860	41.171
	rs6882345	A	G	0.044	0.633	52.011
	rs6938647	C	A	-0.048	0.782	31.837
	rs7139079	A	G	-0.029	0.593	24.406
	rs7707394	A	G	0.029	0.357	22.572
	rs7750288	G	A	0.025	0.285	12.918
	rs9304381	T	C	0.070	0.818	53.251
	rs9616847	T	A	0.024	0.388	15.203
	rs964184	C	G	-0.139	0.867	125.990
**Exposure**	**SNP**	**effect allele**	**other allele**	**beta**	**eaf**	**F**
	rs1077835	G	A	-0.100	0.220	148.246
	rs12226389	C	T	0.059	0.186	40.491
	rs1260326	C	T	0.066	0.604	128.279
	rs1532085	G	A	0.086	0.614	212.433
	rs174564	G	A	0.371	0.347	3757.161
**Omega-6/Omega-3**	rs195445	T	C	0.034	0.688	27.801
	rs4860987	T	A	-0.041	0.259	28.174
	rs583609	C	T	0.043	0.353	48.405
	rs58542926	T	C	0.144	0.074	50.107
	rs62466318	T	C	0.060	0.204	47.755
	rs6693447	G	T	-0.025	0.462	19.707
	rs673335	C	T	0.061	0.160	34.243
	rs7916868	T	A	-0.028	0.505	25.322
	rs8074191	C	T	0.028	0.756	13.612
	rs9332238	A	G	-0.035	0.198	15.443
	rs964184	C	G	0.075	0.867	37.808
	rs9947684	G	A	-0.028	0.654	19.877
**Exposure**	**SNP**	**effect allele**	**other allele**	**beta**	**eaf**	**F**
	rs10455872	G	A	0.138	0.079	51.721
	rs10733306	T	C	0.022	0.460	15.067
	rs10773049	C	T	0.022	0.393	14.617
	rs11429307	GT	G	-0.045	0.191	24.112
	rs11508026	T	C	0.027	0.432	22.793
	rs1168030	T	C	-0.049	0.645	65.042
	rs11976955	G	C	-0.026	0.314	15.547
	rs12419462	A	G	-0.029	0.229	12.950
	rs1260326	C	T	0.110	0.604	353.179
	rs1316753	C	G	0.023	0.395	15.168
	rs13389219	T	C	0.038	0.393	42.646
	rs1471251	T	A	-0.026	0.397	20.229
**Omega-6/Total FAs**	rs1546224	T	C	-0.024	0.309	12.953
	rs1736070	T	C	0.026	0.666	16.598
	rs174528	C	T	0.025	0.377	17.799
	rs2068888	A	G	0.026	0.450	20.688
	rs261290	C	T	0.062	0.655	99.033
	rs28601761	G	C	0.085	0.420	216.084
	rs295268	C	T	0.028	0.256	14.524

	rs2972140	C	T	-0.034	0.651	30.702
	rs328	G	C	0.138	0.100	79.104
	rs3812316	G	C	0.099	0.129	63.962
	rs4665710	C	A	-0.047	0.793	30.998
	rs473224	G	T	0.060	0.854	27.933
	rs499765	G	C	0.026	0.339	16.361
	rs58489806	T	C	0.070	0.086	15.630
	rs6073958	C	T	-0.047	0.199	29.033
	rs6658257	G	A	-0.023	0.602	15.235
	rs684773	C	A	-0.035	0.767	19.609
	rs6905288	A	G	-0.026	0.569	20.108
	rs7402939	C	T	-0.024	0.624	15.199
	rs964184	C	G	0.136	0.867	125.496
	rs9908820	G	A	-0.028	0.729	15.445
**Exposure**	**SNP**	**effect allele**	**other allele**	**beta**	**eaf**	**F**
	rs1002687	A	G	0.097	0.645	244.705
	rs102275	C	T	-0.097	0.350	246.511
	rs11789603	T	C	0.049	0.109	10.984
	rs1260326	C	T	-0.077	0.604	169.006
	rs12899324	T	C	-0.025	0.267	11.822
	rs13108218	G	A	-0.035	0.615	32.855
	rs2126259	C	T	0.085	0.899	29.415
	rs2326077	T	C	-0.029	0.663	20.813
	rs261290	C	T	-0.112	0.655	318.181
	rs2737245	T	G	-0.029	0.279	16.764
	rs2740488	C	A	-0.049	0.265	44.727
	rs34955778	C	T	-0.030	0.420	26.222
	rs3764261	A	C	0.056	0.324	74.919
	rs3770586	T	C	-0.024	0.484	17.726
	rs3822855	T	G	0.024	0.401	17.044
	rs3843482	G	T	0.042	0.374	47.549
**Polyunsaturated FAs**	rs4299376	T	G	-0.032	0.676	23.837
	rs4766578	A	T	0.027	0.503	22.621
	rs4860948	A	T	0.034	0.244	19.481
	rs553427	T	C	0.029	0.517	26.988
	rs55747707	A	G	-0.061	0.204	48.992
	rs58542926	T	C	-0.153	0.074	55.225
	rs633695	G	A	0.085	0.292	151.281
	rs660240	C	T	0.059	0.785	49.490
	rs6602911	T	C	0.028	0.360	21.111
	rs672889	G	T	0.074	0.860	39.792
	rs6882345	A	G	0.045	0.633	53.882
	rs6938647	C	A	-0.047	0.782	30.795
	rs72997616	A	C	-0.066	0.094	15.675
	rs7707394	A	G	0.026	0.357	18.482
	rs7970695	A	G	-0.031	0.621	27.270
	rs9304381	T	C	0.073	0.818	59.092
	rs9616847	T	A	0.023	0.388	14.378
	rs964184	C	G	-0.150	0.867	149.554
**Exposure**	**SNP**	**effect allele**	**other allele**	**beta**	**eaf**	**F**
	rs10020067	A	C	-0.027	0.381	20.863
	rs10455872	G	A	0.132	0.079	46.851
	rs10746732	A	G	-0.028	0.778	11.740
	rs11429307	GT	G	-0.040	0.191	19.253
	rs11644601	C	T	0.053	0.297	60.926
	rs1168041	C	T	-0.036	0.655	32.957
	rs1180384	C	T	-0.026	0.302	14.918
	rs12419462	A	G	-0.031	0.229	14.852
	rs1260326	C	T	0.090	0.604	238.869
	rs13389219	T	C	0.035	0.393	36.370
**Polyunsaturated FAs/total FAs**	rs174564	G	A	-0.122	0.347	389.611
	rs2081194	C	G	0.028	0.601	21.962
	rs261290	C	T	0.028	0.655	20.412
	rs28601761	G	C	0.062	0.420	114.981
	rs2972140	C	T	-0.034	0.651	30.396
	rs328	G	C	0.136	0.100	77.212
	rs3812316	G	C	0.078	0.129	39.135
	rs4871624	G	T	-0.024	0.287	12.615
	rs56156922	C	T	0.027	0.324	17.602
	rs6073958	C	T	-0.053	0.199	36.528
	rs632057	G	T	0.028	0.628	21.468
	rs673548	A	G	0.043	0.206	25.131
	rs6905288	A	G	-0.023	0.569	15.628
	rs7402939	C	T	-0.024	0.624	15.351
	rs752446	A	G	-0.026	0.375	18.812
	rs7924036	T	G	0.037	0.504	42.697
	rs9419746	T	C	-0.029	0.213	12.050
	rs964184	C	G	0.115	0.867	89.196
	rs9908820	G	A	-0.029	0.729	16.095
**Exposure**	**SNP**	**effect allele**	**other allele**	**beta**	**eaf**	**F**
	rs1002687	A	G	0.082	0.645	172.773
	rs102275	C	T	-0.033	0.350	27.457
	rs10455872	G	A	-0.135	0.079	46.993
	rs10504255	A	G	-0.028	0.663	19.189
	rs10810374	C	A	0.026	0.249	11.220
	rs11076175	G	A	-0.039	0.179	15.615
	rs11429307	GT	G	0.034	0.191	13.290
	rs1260326	C	T	-0.106	0.604	314.186
	rs13108218	G	A	-0.028	0.615	20.571
	rs13389219	T	C	-0.030	0.393	24.609
	rs139315015	G	A	-0.084	0.105	30.406
	rs2126259	C	T	0.057	0.899	13.025
	rs2156552	T	A	0.051	0.822	27.108
	rs2478236	A	G	-0.025	0.404	17.613
	rs261290	C	T	-0.101	0.655	250.059
	rs2740488	C	A	-0.036	0.265	24.153
	rs28383314	C	T	0.039	0.623	39.754
**Saturated FAs**	rs28601761	G	C	-0.091	0.420	232.156
	rs3846661	G	A	0.026	0.398	19.405
	rs4564803	T	G	-0.049	0.228	36.784
	rs4704834	G	A	0.037	0.644	35.300
	rs55747707	A	G	-0.070	0.204	61.462
	rs60960031	A	G	-0.025	0.403	17.830
	rs632057	G	T	-0.028	0.628	21.280
	rs633695	G	A	0.073	0.292	109.744
	rs660240	C	T	0.032	0.785	13.739
	rs6602911	T	C	0.026	0.360	17.723
	rs6854749	T	A	0.032	0.198	12.628
	rs7973253	G	A	0.028	0.367	20.449
	rs8107974	T	A	-0.117	0.076	32.366
	rs900048	T	C	0.026	0.710	14.173
	rs9616847	T	A	0.023	0.388	14.412
	rs964184	C	G	-0.145	0.867	135.710
**Exposure**	**SNP**	**effect allele**	**other allele**	**beta**	**eaf**	**F**
	rs1002687	A	G	0.096	0.645	234.256
	rs102275	C	T	-0.025	0.350	15.925
	rs10455872	G	A	-0.133	0.079	45.024
	rs11076175	G	A	-0.044	0.179	20.079
	rs1128249	T	G	-0.029	0.392	22.644
	rs11429307	GT	G	0.035	0.191	13.694
	rs1260326	C	T	-0.106	0.604	310.259
	rs13108218	G	A	-0.035	0.615	33.164
	rs1471251	T	A	0.026	0.397	19.083
	rs2000999	A	G	0.031	0.189	10.550
	rs2126259	C	T	0.067	0.899	17.992
	rs2156552	T	A	0.057	0.822	33.613
	rs2326077	T	C	-0.029	0.663	20.655

	rs2478236	A	G	-0.025	0.404	16.732
	rs261290	C	T	-0.101	0.655	251.260
	rs2721961	G	T	-0.028	0.281	15.245
	rs2740488	C	A	-0.040	0.265	29.244
**Total FAs**	rs28383314	C	T	0.043	0.623	50.348
	rs28601761	G	C	-0.092	0.420	236.459
	rs328	G	C	-0.096	0.100	36.012
	rs3846663	T	C	0.032	0.374	27.189
	rs4299376	T	G	-0.024	0.676	13.039
	rs4564803	T	G	-0.062	0.228	57.200
	rs4704834	G	A	0.045	0.644	50.569
	rs4860948	A	T	0.028	0.244	12.552
	rs55747707	A	G	-0.080	0.204	80.456
	rs58542926	T	C	-0.140	0.074	44.700
	rs60960031	A	G	-0.028	0.403	22.042
	rs632057	G	T	-0.028	0.628	20.692
	rs633695	G	A	0.076	0.292	117.251
	rs660240	C	T	0.041	0.785	22.726
	rs6602911	T	C	0.029	0.360	22.039
	rs7973253	G	A	0.027	0.367	19.017
	rs822928	C	A	0.024	0.530	17.038
	rs900048	T	C	0.027	0.710	14.564
	rs9616847	T	A	0.024	0.388	15.411
	rs964184	C	G	-0.178	0.867	203.600
**Exposure**	**SNP**	**effect allele**	**other allele**	**beta**	**eaf**	**F**
	rs10455872	G	A	0.100	0.079	27.402
	rs1077835	G	A	0.042	0.220	27.385
	rs11644601	C	T	0.093	0.297	196.742
	rs12226389	C	T	-0.059	0.186	40.899
	rs1260326	C	T	0.040	0.604	46.651
	rs13389219	T	C	0.022	0.393	14.508
	rs1532085	G	A	-0.039	0.614	44.445
	rs174564	G	A	-0.341	0.347	3240.374
	rs2081194	C	G	0.027	0.601	20.860
	rs272891	T	G	0.033	0.707	24.558
	rs2972140	C	T	-0.030	0.651	24.887
**Degree of unsaturation**	rs328	G	C	0.119	0.100	60.547
	rs4296389	T	C	0.029	0.250	15.075
	rs459193	G	A	-0.025	0.747	11.780
	rs498936	A	T	-0.059	0.158	32.591
	rs58542926	T	C	-0.064	0.074	10.248
	rs6073958	C	T	-0.056	0.199	40.974
	rs625384	T	C	-0.027	0.302	16.389
	rs660240	C	T	0.030	0.785	13.334
	rs7234218	G	A	0.031	0.590	28.537
	rs73045691	A	G	0.027	0.304	15.573
	rs7924036	T	G	0.046	0.504	67.867
	rs964184	C	G	0.057	0.867	22.633
**Exposure**	**SNP**	**effect allele**	**other allele**	**beta**	**eaf**	**F**
**Average number of methylene groups in a fatty acid chain**	rs12226389	C	T	0.089	0.189	11.933
	rs174546	T	C	0.238	0.384	211.453
**Exposure**	**SNP**	**effect allele**	**other allele**	**beta**	**eaf**	**F**
	rs11644601	C	T	0.073	0.278	13.136
**Average number of double bonds in a fatty acid chain**	rs12226389	C	T	-0.093	0.185	11.348
	rs174546	T	C	-0.293	0.403	281.956
**Exposure**	**SNP**	**effect allele**	**other allele**	**beta**	**eaf**	**F**
	rs1077835	G	A	0.128	0.250	29.433
	rs1260326	C	T	-0.078	0.637	17.444
**Other polyunsaturated fatty acids than 18:2**	rs174418	C	T	-0.086	0.562	23.031
	rs174528	C	T	-0.388	0.413	518.681
	rs3019558	A	G	-0.076	0.433	17.286
	rs3741298	T	C	-0.088	0.770	12.993
	rs4382917	A	G	0.104	0.247	16.119
	rs754382	G	A	0.083	0.724	14.325
