# Supplementary material for: Genetically Predicted Gut Microbiota Mediate the Association Between Fatty Acids and Intrahepatic Cholestasis of Pregnancy: A Mendelian Randomization Analysis
Source: Food Sci Nutr. 2024 Dec 30;13(1):e4683. doi: 10.1002/fsn3.4683 (PMC11717022; doi:10.1002/fsn3.4683)
Supplement: Supplementary file 3 — Table S3. SNPs used for MR analysis in gut bacteria and ICP. [file FSN3-13-e4683-s004.pdf]

Supplementary Table 4: SNPs used for MR analysis in gut bacteria and intrahepatic cholestasis of pregnancy

Exposure	SNP	effect allele	other allele	beta	eaf	F
**An181**	rs6697926	C	T	0.048	0.361	13.768
**Exposure**	**SNP**	**effect allele**	**other allele**	**beta**	**eaf**	**F**
	rs3774209	A	C	-0.014	0.394	11.451
**Bacillaceae A**	rs6763811	C	T	-0.017	0.319	14.187
	rs9979304	G	A	-0.013	0.438	10.423
**Exposure**	**SNP**	**effect allele**	**other allele**	**beta**	**eaf**	**F**
**Bacillales A**	rs9836224	T	C	-0.014	0.319	13.957
**Exposure**	**SNP**	**effect allele**	**other allele**	**beta**	**eaf**	**F**
**Bin127**	rs7337561	T	G	-0.029	0.297	10.621
**Exposure**	**SNP**	**effect allele**	**other allele**	**beta**	**eaf**	**F**
	rs2999156	C	G	-0.072	0.412	11.775
**CAG-177 sp003538135**	rs34966720	C	G	-0.083	0.454	15.545
	rs7197468	G	T	-0.067	0.428	10.092
	rs9375059	G	A	0.069	0.493	11.037
**Exposure**	**SNP**	**effect allele**	**other allele**	**beta**	**eaf**	**F**
**Collinsella**	rs4723822	T	C	0.062	0.289	10.404
**Exposure**	**SNP**	**effect allele**	**other allele**	**beta**	**eaf**	**F**
**Coprobacillus cateniformis**	rs5012948	T	C	-0.050	0.423	10.260
**Exposure**	**SNP**	**effect allele**	**other allele**	**beta**	**eaf**	**F**
**Coprobacillus**	rs10082951	T	C	-0.055	0.423	10.398
**Exposure**	**SNP**	**effect allele**	**other allele**	**beta**	**eaf**	**F**
	rs10831606	G	A	-0.026	0.401	11.340
**Dokdonella**	rs1475166	C	T	0.034	0.226	11.274
	rs475543	A	G	0.025	0.432	12.067
	rs7785032	C	T	-0.027	0.397	12.777
**Exposure**	**SNP**	**effect allele**	**other allele**	**beta**	**eaf**	**F**
	rs1914544	C	G	-0.052	0.363	10.423
**Enteroscipio**	rs2062902	T	C	0.060	0.397	14.511
	rs740365	T	C	0.049	0.404	10.200
**Exposure**	**SNP**	**effect allele**	**other allele**	**beta**	**eaf**	**F**
**Faecalicatena sp000364245**	rs11165411	A	G	-0.035	0.382	15.170
	rs1481716	A	C	-0.028	0.399	10.255
**Exposure**	**SNP**	**effect allele**	**other allele**	**beta**	**eaf**	**F**
**Faecalicatena torques**	rs1030431	A	G	-0.080	0.306	11.714
	rs3770594	G	C	0.069	0.466	12.014
**Exposure**	**SNP**	**effect allele**	**other allele**	**beta**	**eaf**	**F**
**Gluconobacter**	rs6900735	A	G	0.023	0.357	10.061
**Exposure**	**SNP**	**effect allele**	**other allele**	**beta**	**eaf**	**F**
	rs138657	A	G	0.050	0.495	10.683
**Lactobacillus B**	rs1560481	A	G	0.052	0.413	10.406
	rs2143139	G	C	-0.050	0.451	10.180
	rs2648545	C	T	-0.059	0.337	11.082
	rs7350155	T	C	0.059	0.302	10.144
**Exposure**	**SNP**	**effect allele**	**other allele**	**beta**	**eaf**	**F**
**Lawsonibacter sp002161175**	rs582100	G	C	0.037	0.465	14.693
	rs787419	T	A	0.035	0.374	11.482
**Exposure**	**SNP**	**effect allele**	**other allele**	**beta**	**eaf**	**F**
	rs259848	C	T	0.030	0.417	10.808
**Morganella**	rs6500635	G	A	0.030	0.434	11.340
	rs7378239	A	T	-0.030	0.421	10.833
**Exposure**	**SNP**	**effect allele**	**other allele**	**beta**	**eaf**	**F**
	rs1024200	G	A	-0.065	0.475	13.049
	rs2055258	T	C	0.060	0.391	10.629
**Parabacteroides johnsonii**	rs6449609	A	T	0.060	0.384	10.989
**Exposure**	**SNP**	**effect allele**	**other allele**	**beta**	**eaf**	**F**

	rs12673506	A	G	0.102	0.201	10.728
	rs2425862	A	G	0.066	0.484	10.492
**Parabacteroides**	rs2791577	T	A	0.066	0.414	10.587
	rs4843380	C	G	-0.065	0.461	10.793
	rs7073854	G	T	0.068	0.396	10.686
	rs9290864	G	A	0.068	0.368	10.398
**Exposure**	**SNP**	**effect allele**	**other allele**	**beta**	**eaf**	**F**
**Parachlamydiales**	rs2757529	C	T	-0.021	0.436	15.130
**Exposure**	**SNP**	**effect allele**	**other allele**	**beta**	**eaf**	**F**
**Planococcaceae**	rs9836224	T	C	-0.016	0.319	14.882
**Exposure**	**SNP**	**effect allele**	**other allele**	**beta**	**eaf**	**F**
	rs4409465	A	T	0.031	0.308	10.310
**Ruminococcus**	rs4739376	C	T	0.026	0.463	10.058
	rs696938	T	C	-0.026	0.447	10.325
**Exposure**	**SNP**	**effect allele**	**other allele**	**beta**	**eaf**	**F**
	rs10033651	T	C	-0.017	0.461	12.168
	rs2385502	T	C	-0.017	0.413	11.641
**SAR324**	rs4698504	T	C	0.016	0.384	10.439
	rs533359	G	A	0.016	0.479	11.749
	rs8006853	T	C	0.016	0.426	10.210
**Exposure**	**SNP**	**effect allele**	**other allele**	**beta**	**eaf**	**F**
**Succinivibrio**	rs17023722	C	T	0.052	0.315	10.296
**Exposure**	**SNP**	**effect allele**	**other allele**	**beta**	**eaf**	**F**
**Syntrophorhabdaceae**	rs2913633	C	T	0.021	0.498	10.555
	rs6468721	C	T	0.029	0.265	11.624
**Exposure**	**SNP**	**effect allele**	**other allele**	**beta**	**eaf**	**F**
	rs11924583	G	A	0.067	0.369	11.422
	rs12673506	A	G	0.093	0.201	10.087
**Tannerellaceae**	rs2425862	A	G	0.063	0.484	10.774
	rs2791577	T	A	0.061	0.414	10.055
	rs4843380	C	G	-0.061	0.461	10.827
	rs7073854	G	T	0.063	0.396	10.540
