# Supplementary material for: Genetically Predicted Gut Microbiota Mediate the Association Between Fatty Acids and Intrahepatic Cholestasis of Pregnancy: A Mendelian Randomization Analysis
Source: Food Sci Nutr. 2024 Dec 30;13(1):e4683. doi: 10.1002/fsn3.4683 (PMC11717022; doi:10.1002/fsn3.4683)
Supplement: Supplementary file 4 — Table S4. SNPs used for MR analysis FAs and gut bacteria. [file FSN3-13-e4683-s003.pdf]

Supplementary Table 3: SNPs used for MR analysis in fatty acids and gut bacteria

Outcome:

Exposure	SNP	effect allele	other allele	beta	eaf	F
	rs11122450	G	T	0.022	0.611	14.390
	rs112875651	A	G	-0.049	0.392	69.878
	rs11681659	T	C	-0.031	0.716	20.306
	rs12226389	C	T	-0.047	0.186	25.495
	rs1260326	C	T	-0.048	0.604	68.252
	rs12872990	G	A	-0.025	0.707	12.554
	rs12914626	T	C	-0.061	0.702	83.002
	rs1560390	C	T	-0.050	0.220	37.751
	rs16940904	T	C	-0.039	0.227	24.077
	rs174564	G	A	-0.290	0.347	2309.862
	rs261291	C	T	0.114	0.356	354.631
	rs273912	T	G	0.026	0.707	15.165
	rs2807967	C	T	0.025	0.718	13.483
	rs2853968	G	A	0.025	0.403	18.107
	rs312939	A	G	0.039	0.752	27.128
	rs3764261	A	C	0.039	0.324	37.635
	rs4860987	T	A	0.038	0.259	25.066
	rs525028	A	G	-0.030	0.709	20.038
	rs55891451	C	A	0.032	0.202	13.359
	rs58542926	T	C	-0.120	0.074	35.310
	rs638714	T	G	-0.038	0.346	37.450
	rs660240	C	T	0.043	0.785	27.612
	rs673335	C	T	-0.057	0.160	30.450
	rs72789541	A	T	-0.080	0.296	144.223
	rs73045691	A	G	0.029	0.304	18.001
	rs7570469	T	C	-0.023	0.669	13.741
	rs78689694	C	G	0.041	0.133	11.403
	rs7924036	T	G	0.035	0.504	39.059
	rs9304381	T	C	0.050	0.818	28.255
	rs9987289	G	A	0.056	0.909	11.317

Exposure	SNP	effect allele	other allele	beta	eaf	F
	rs10733306	T	C	0.025	0.460	19.490
	rs10806735	C	G	0.035	0.696	27.652
	rs11030088	A	G	-0.031	0.251	17.130
	rs115478735	T	A	0.032	0.183	11.687
	rs1260326	C	T	0.082	0.604	192.571
	rs13179413	T	C	-0.029	0.285	17.469
	rs13389219	T	C	0.028	0.393	22.955
	rs17244834	T	A	0.033	0.374	29.989
	rs174567	G	A	0.186	0.348	917.270
	rs199607859	T	G	0.025	0.595	18.694
	rs2180725	C	T	-0.026	0.232	11.140
	rs2576362	T	G	0.027	0.255	13.288
	rs2835028	C	A	0.024	0.412	16.615
	rs2843126	G	A	0.024	0.518	17.450
	rs28601761	G	C	0.066	0.420	128.829
	rs2927468	G	A	0.024	0.523	17.011
	rs2972143	G	A	-0.028	0.646	20.659
	rs34062580	A	G	0.066	0.130	28.280
	rs34620476	A	C	0.031	0.432	28.623
	rs4074872	A	G	0.032	0.234	14.217
	rs4299376	T	G	-0.028	0.676	18.434
	rs4810479	T	C	0.039	0.750	26.560
	rs58365744	C	A	-0.027	0.219	10.456

	rs6683445	A	C	-0.024	0.532	18.123
	rs738408	T	C	0.035	0.217	18.175
	rs7819706	G	A	0.068	0.118	25.118
	rs964184	C	G	0.044	0.867	13.071
	rs9908820	G	A	-0.025	0.729	12.674

Exposure	SNP	effect allele	other allele	beta	eaf	F
	rs10096633	T	C	-0.042	0.124	10.573
	rs10455872	G	A	-0.062	0.079	10.312
	rs11242109	T	G	0.023	0.479	17.492
	rs112875651	A	G	-0.087	0.392	216.222
	rs11681659	T	C	-0.026	0.716	14.871
	rs12037485	T	C	0.022	0.460	15.666
	rs12226389	C	T	-0.054	0.186	33.300
	rs1260326	C	T	-0.083	0.604	202.365
	rs16940904	T	C	-0.035	0.227	18.517
	rs174564	G	A	-0.337	0.347	3100.766
	rs2187375	G	A	0.052	0.823	29.879
	rs2247056	C	T	0.036	0.703	28.301
	rs2288912	G	C	0.027	0.496	22.357
	rs261290	C	T	-0.113	0.655	332.724
	rs312939	A	G	0.034	0.752	20.812
	rs4000713	A	G	-0.028	0.295	17.593
	rs4860987	T	A	0.045	0.259	34.034
	rs5112	G	C	0.050	0.534	67.570
	rs55891451	C	A	0.033	0.202	14.482
	rs583609	C	T	-0.071	0.353	135.118
	rs58542926	T	C	-0.173	0.074	72.347
	rs6129624	A	G	-0.024	0.335	14.469
	rs62466318	T	C	-0.072	0.204	68.811
	rs633695	G	A	0.085	0.292	156.511
	rs660240	C	T	0.037	0.785	19.985
	rs6602911	T	C	0.023	0.360	14.587
	rs673335	C	T	-0.068	0.160	43.212
	rs6882345	A	G	0.027	0.633	20.800
	rs72789541	A	T	-0.083	0.296	153.952
	rs7924036	T	G	0.022	0.504	15.779
	rs7970695	A	G	-0.024	0.621	15.608
	rs964184	C	G	-0.118	0.867	94.528
	rs9987289	G	A	0.054	0.909	10.314

Exposure	SNP	effect allele	other allele	beta	eaf	F
	rs10075801	G	A	-0.024	0.485	17.242
	rs1077835	G	A	-0.100	0.220	148.246
	rs112875651	A	G	0.072	0.392	145.224
	rs12226389	C	T	0.059	0.186	40.491
	rs1260326	C	T	0.066	0.604	128.279
	rs12976395	C	G	-0.027	0.505	20.304
	rs1532085	G	A	0.086	0.614	212.433
	rs174564	G	A	0.371	0.347	3757.161
	rs195445	T	C	0.034	0.688	27.801
	rs2463523	C	A	0.040	0.235	25.591
	rs4000713	A	G	0.030	0.295	20.008
	rs4860987	T	A	-0.041	0.259	28.174
	rs583609	C	T	0.043	0.353	48.405
	rs58542926	T	C	0.144	0.074	50.107
	rs62466318	T	C	0.060	0.204	47.755
	rs6693447	G	T	-0.025	0.462	19.707
	rs673335	C	T	0.061	0.160	34.243
	rs72789541	A	T	0.091	0.296	180.392

Exposure	SNP	effect allele	other allele	beta	eaf	F
	rs7916868	T	A	-0.028	0.505	25.322
	rs8074191	C	T	0.028	0.756	13.612
	rs9332238	A	G	-0.035	0.198	15.443
	rs964184	C	G	0.075	0.867	37.808
	rs9947684	G	A	-0.028	0.654	19.877

Exposure	SNP	effect allele	other allele	beta	eaf	F
	rs10455872	G	A	0.138	0.079	51.721
	rs10733306	T	C	0.022	0.460	15.067
	rs10773049	C	T	0.022	0.393	14.617
	rs11508026	T	C	0.027	0.432	22.793
	rs1168030	T	C	-0.049	0.645	65.042
	rs11976955	G	C	-0.026	0.314	15.547
	rs12419462	A	G	-0.029	0.229	12.950
	rs1260326	C	T	0.110	0.604	353.179
	rs1316753	C	G	0.023	0.395	15.168
	rs13389219	T	C	0.038	0.393	42.646
	rs1471251	T	A	-0.026	0.397	20.229
	rs1546224	T	C	-0.024	0.309	12.953
	rs1561748	C	G	0.049	0.267	41.124
	rs1736070	T	C	0.026	0.666	16.598
	rs174528	C	T	0.025	0.377	17.799
	rs199607859	T	G	0.032	0.595	29.684
	rs2068888	A	G	0.026	0.450	20.688
	rs261290	C	T	0.062	0.655	99.033
	rs28601761	G	C	0.085	0.420	216.084
	rs28752523	T	C	-0.035	0.194	15.527
	rs295268	C	T	0.028	0.256	14.524
	rs2972140	C	T	-0.034	0.651	30.702
	rs328	G	C	0.138	0.100	79.104
	rs3812316	G	C	0.099	0.129	63.962
	rs3860846	T	C	-0.030	0.276	18.010
	rs4665710	C	A	-0.047	0.793	30.998
	rs473224	G	T	0.060	0.854	27.933
	rs58489806	T	C	0.070	0.086	15.630
	rs6073958	C	T	-0.047	0.199	29.033
	rs6658257	G	A	-0.023	0.602	15.235
	rs684773	C	A	-0.035	0.767	19.609
	rs6905288	A	G	-0.026	0.569	20.108
	rs7402939	C	T	-0.024	0.624	15.199
	rs964184	C	G	0.136	0.867	125.496
	rs9908820	G	A	-0.028	0.729	15.445

Exposure	SNP	effect allele	other allele	beta	eaf	F
	rs12226389	C	T	0.089	0.189	11.933
	rs174546	T	C	0.238	0.384	211.453

Exposure	SNP	effect allele	other allele	beta	eaf	F
	rs11644601	C	T	0.073	0.278	13.136
	rs12226389	C	T	-0.093	0.185	11.348
	rs174546	T	C	-0.293	0.403	281.956

Exposure	SNP	effect allele	other allele	beta	eaf	F
	rs11122450	G	T	0.022	0.611	14.390
	rs112875651	A	G	-0.049	0.392	69.878
	rs11681659	T	C	-0.031	0.716	20.306
	rs12226389	C	T	-0.047	0.186	25.495
	rs1260326	C	T	-0.048	0.604	68.252
	rs12872990	G	A	-0.025	0.707	12.554
	rs12914626	T	C	-0.061	0.702	83.002
	rs1560390	C	T	-0.050	0.220	37.751

	rs16940904	T	C	-0.039	0.227	24.077
	rs174564	G	A	-0.290	0.347	2309.862
	rs261291	C	T	0.114	0.356	354.631
	rs273912	T	G	0.026	0.707	15.165
	rs2807967	C	T	0.025	0.718	13.483
	rs2853968	G	A	0.025	0.403	18.107
	rs312939	A	G	0.039	0.752	27.128
	rs3764261	A	C	0.039	0.324	37.635
	rs4860987	T	A	0.038	0.259	25.066
	rs525028	A	G	-0.030	0.709	20.038
	rs55891451	C	A	0.032	0.202	13.359
	rs58542926	T	C	-0.120	0.074	35.310
	rs638714	T	G	-0.038	0.346	37.450
	rs660240	C	T	0.043	0.785	27.612
	rs673335	C	T	-0.057	0.160	30.450
	rs72789541	A	T	-0.080	0.296	144.223
	rs73045691	A	G	0.029	0.304	18.001
	rs7570469	T	C	-0.023	0.669	13.741
	rs78689694	C	G	0.041	0.133	11.403
	rs7924036	T	G	0.035	0.504	39.059
	rs9304381	T	C	0.050	0.818	28.255
	rs9987289	G	A	0.056	0.909	11.317
**Exposure**	**SNP**	**effect allele**	**other allele**	**beta**	**eaf**	**F**
	rs10733306	T	C	0.025	0.460	19.490
	rs10806735	C	G	0.035	0.696	27.652
	rs11030088	A	G	-0.031	0.251	17.130
	rs115478735	T	A	0.032	0.183	11.687
	rs1260326	C	T	0.082	0.604	192.571
	rs13179413	T	C	-0.029	0.285	17.469
	rs13389219	T	C	0.028	0.393	22.955
	rs17244834	T	A	0.033	0.374	29.989
	rs174567	G	A	0.186	0.348	917.270
	rs199607859	T	G	0.025	0.595	18.694
	rs2180725	C	T	-0.026	0.232	11.140
	rs2576362	T	G	0.027	0.255	13.288
	rs2835028	C	A	0.024	0.412	16.615
	rs2843126	G	A	0.024	0.518	17.450
	rs28601761	G	C	0.066	0.420	128.829
	rs2927468	G	A	0.024	0.523	17.011
	rs2972143	G	A	-0.028	0.646	20.659
	rs34062580	A	G	0.066	0.130	28.280
	rs34620476	A	C	0.031	0.432	28.623
	rs4074872	A	G	0.032	0.234	14.217
	rs4299376	T	G	-0.028	0.676	18.434
	rs4810479	T	C	0.039	0.750	26.560
	rs58365744	C	A	-0.027	0.219	10.456
	rs6683445	A	C	-0.024	0.532	18.123
	rs738408	T	C	0.035	0.217	18.175
	rs7819706	G	A	0.068	0.118	25.118
	rs964184	C	G	0.044	0.867	13.071
	rs9908820	G	A	-0.025	0.729	12.674
**Exposure**	**SNP**	**effect allele**	**other allele**	**beta**	**eaf**	**F**
	rs10096633	T	C	-0.042	0.124	10.573
	rs10455872	G	A	-0.062	0.079	10.312
	rs11242109	T	G	0.023	0.479	17.492
	rs112875651	A	G	-0.087	0.392	216.222
	rs11681659	T	C	-0.026	0.716	14.871
	rs12037485	T	C	0.022	0.460	15.666

Exposure	SNP	effect allele	other allele	beta	eaf	F
	rs12226389	C	T	-0.054	0.186	33.300
	rs1260326	C	T	-0.083	0.604	202.365
	rs16940904	T	C	-0.035	0.227	18.517
	rs174564	G	A	-0.337	0.347	3100.766
	rs2187375	G	A	0.052	0.823	29.879
	rs2247056	C	T	0.036	0.703	28.301
	rs2288912	G	C	0.027	0.496	22.357
	rs261290	C	T	-0.113	0.655	332.724
	rs312939	A	G	0.034	0.752	20.812
	rs4000713	A	G	-0.028	0.295	17.593
	rs4860987	T	A	0.045	0.259	34.034
	rs5112	G	C	0.050	0.534	67.570
	rs55891451	C	A	0.033	0.202	14.482
	rs583609	C	T	-0.071	0.353	135.118
	rs58542926	T	C	-0.173	0.074	72.347
	rs6129624	A	G	-0.024	0.335	14.469
	rs62466318	T	C	-0.072	0.204	68.811
	rs633695	G	A	0.085	0.292	156.511
	rs660240	C	T	0.037	0.785	19.985
	rs6602911	T	C	0.023	0.360	14.587
	rs673335	C	T	-0.068	0.160	43.212
	rs6882345	A	G	0.027	0.633	20.800
	rs72789541	A	T	-0.083	0.296	153.952
	rs7924036	T	G	0.022	0.504	15.779
	rs7970695	A	G	-0.024	0.621	15.608
	rs964184	C	G	-0.118	0.867	94.528
	rs9987289	G	A	0.054	0.909	10.314
**Exposure**	**SNP**	**effect allele**	**other allele**	**beta**	**eaf**	**F**
	rs10075801	G	A	-0.024	0.485	17.242
	rs1077835	G	A	-0.100	0.220	148.246
	rs112875651	A	G	0.072	0.392	145.224
	rs12226389	C	T	0.059	0.186	40.491
	rs1260326	C	T	0.066	0.604	128.279
	rs12976395	C	G	-0.027	0.505	20.304
	rs1532085	G	A	0.086	0.614	212.433
	rs174564	G	A	0.371	0.347	3757.161
	rs195445	T	C	0.034	0.688	27.801
	rs2463523	C	A	0.040	0.235	25.591
	rs4000713	A	G	0.030	0.295	20.008
	rs4860987	T	A	-0.041	0.259	28.174
	rs583609	C	T	0.043	0.353	48.405
	rs58542926	T	C	0.144	0.074	50.107
	rs62466318	T	C	0.060	0.204	47.755
	rs6693447	G	T	-0.025	0.462	19.707
	rs673335	C	T	0.061	0.160	34.243
	rs72789541	A	T	0.091	0.296	180.392
	rs7916868	T	A	-0.028	0.505	25.322
	rs8074191	C	T	0.028	0.756	13.612
	rs9332238	A	G	-0.035	0.198	15.443
	rs964184	C	G	0.075	0.867	37.808
	rs9947684	G	A	-0.028	0.654	19.877
**Exposure**	**SNP**	**effect allele**	**other allele**	**beta**	**eaf**	**F**
	rs10455872	G	A	0.138	0.079	51.721
	rs10733306	T	C	0.022	0.460	15.067
	rs10773049	C	T	0.022	0.393	14.617
	rs11508026	T	C	0.027	0.432	22.793
	rs1168030	T	C	-0.049	0.645	65.042
	rs11976955	G	C	-0.026	0.314	15.547

	rs12419462	A	G	-0.029	0.229	12.950
	rs1260326	C	T	0.110	0.604	353.179
	rs1316753	C	G	0.023	0.395	15.168
	rs13389219	T	C	0.038	0.393	42.646
	rs1471251	T	A	-0.026	0.397	20.229
	rs1546224	T	C	-0.024	0.309	12.953
	rs1561748	C	G	0.049	0.267	41.124
	rs1736070	T	C	0.026	0.666	16.598
	rs174528	C	T	0.025	0.377	17.799
	rs199607859	T	G	0.032	0.595	29.684
	rs2068888	A	G	0.026	0.450	20.688
	rs261290	C	T	0.062	0.655	99.033
	rs28601761	G	C	0.085	0.420	216.084
	rs28752523	T	C	-0.035	0.194	15.527
	rs295268	C	T	0.028	0.256	14.524
	rs2972140	C	T	-0.034	0.651	30.702
	rs328	G	C	0.138	0.100	79.104
	rs3812316	G	C	0.099	0.129	63.962
	rs3860846	T	C	-0.030	0.276	18.010
	rs4665710	C	A	-0.047	0.793	30.998
	rs473224	G	T	0.060	0.854	27.933
	rs58489806	T	C	0.070	0.086	15.630
	rs6073958	C	T	-0.047	0.199	29.033
	rs6658257	G	A	-0.023	0.602	15.235
	rs684773	C	A	-0.035	0.767	19.609
	rs6905288	A	G	-0.026	0.569	20.108
	rs7402939	C	T	-0.024	0.624	15.199
	rs964184	C	G	0.136	0.867	125.496
	rs9908820	G	A	-0.028	0.729	15.445
**Exposure**	**SNP**	**effect allele**	**other allele**	**beta**	**eaf**	**F**
	rs12226389	C	T	0.089	0.189	11.933
	rs174546	T	C	0.238	0.384	211.453
**Exposure**	**SNP**	**effect allele**	**other allele**	**beta**	**eaf**	**F**
	rs11644601	C	T	0.073	0.278	13.136
	rs12226389	C	T	-0.093	0.185	11.348
	rs174546	T	C	-0.293	0.403	281.956
